# Robust optimization of SWATH-MS workflow for human blood serum proteome analysis using a quality by design approach

**DOI:** 10.1186/s12014-021-09323-z

**Published:** 2021-08-12

**Authors:** Edith Serrano-Blesa, Andrew Porter, Dennis W. Lendrem, Costantino Pitzalis, Anne Barton, Achim Treumann, John D. Isaacs

**Affiliations:** 1 National Institute of Health Research Newcastle Biomedical Research Centre and the Translational and Clinical Research Institute, Newcastle University, Newcastle upon Tyne, UK; 2 Newcastle University Protein and Proteome Facility, Newcastle upon Tyne, UK; 3 Centre for Experimental Medicine and Rheumatology, Queen Mary University of London, London, UK; 4 Versus Arthritis Centre for Genetics and Genomics, Centre for Musculoskeletal Research, Faculty of Biology, Medicine and Health, Manchester Academic Health Science Centre,, The University of Manchester, Manchester, UK; 5 Musculoskeletal Unit, Freeman Hospital, Newcastle upon Tyne NHS Foundation Trust, Newcastle upon Tyne, UK

**Keywords:** SWATH-MS, Proteomic quantification, Design of experiments, Quality by design, Screening, Optimization, Robustness

## Abstract

**Background:**

It is not enough to optimize proteomics assays. It is critical those assays are robust to operating conditions. Without robust assays, proteomic biomarkers are unlikely to translate readily into the clinic. This study outlines a structured approach to the identification of a robust operating window for proteomics assays and applies that method to Sequential Window Acquisition of all Theoretical Spectra Mass Spectroscopy (SWATH-MS).

**Methods:**

We used a sequential quality by design approach exploiting a fractional screening design to first identify critical SWATH-MS parameters, then using response surface methods to identify a robust operating window with good reproducibility, before validating those settings in a separate validation study.

**Results:**

The screening experiment identified two critical SWATH-MS parameters. We modelled the number of proteins and reproducibility as a function of those parameters identifying an operating window permitting robust maximization of the number of proteins quantified in human serum. In a separate validation study, these settings were shown to give good proteome-wide coverage and high quantification reproducibility.

**Conclusions:**

Using design of experiments permits identification of a robust operating window for SWATH-MS. The method gives a good understanding of proteomics assays and greater data-driven confidence in SWATH-MS performance.

**Supplementary Information:**

The online version contains supplementary material available at 10.1186/s12014-021-09323-z.

## Background

In translational medicine it is not enough to optimize proteomics assays. It is critical those assays are robust to operating conditions, transferring readily to other devices operating in other laboratories. Without robust assays, proteomic biomarkers are unlikely to translate readily into the clinic. The human blood proteome is one of the more clinically relevant matrices for biomarker discovery as it can reflect the physiological changes associated with disease. The fact that it not only carries proteins intrinsic to blood but also others, such as messengers between tissues or products of tissue damage, only adds to its importance and complexity [1, 2]. Most pathological alterations do not modify the general blood protein composition but can affect the relative amount of specific proteins. Therefore, methods capable of both qualitative and quantitative analysis are necessary for the in-depth study of the blood proteome [3].

Liquid chromatography–mass spectrometry (LC–MS) has been frequently used for blood proteome analysis because it is able to encompass complex protein mixtures, even though the high dynamic range present in serum remains challenging. Classic approaches allowed for either identification of a high number of proteins (Data-Dependent Acquisition methods or Information-Dependent Acquisition, DDA and IDA respectively) or for accurate quantification of a limited number of proteins (Multiple Reaction Monitoring, MRM) [4]. However, technical improvements have resulted in the creation of Data-Independent Acquisition (DIA) methods which combine DDA coverage and MRM accuracy, removing the randomness of DDA in the selection of peaks for fragmentation while maintaining high sensitivity [5]. Sequential Window Acquisition of all Theoretical Spectra (SWATH) is one of the most commonly used DIA implementations. A SWATH method fragments all the peptide precursors in sequential isolated windows across the m/z range without a pre-selection of the precursor ions, thus increasing the run-to-run reproducibility and allowing for reliable quantification of a high number of proteins [6].

Robust optimization of sample preparation, LC and SWATH acquisition parameters may improve the results in the analysis of complex protein samples by increasing the signal-to-noise ratio of observed transitions and, consequently, the number of peptides and/or proteins per sample that can be identified and/or quantified. Different studies [7–10] have shown an improvement in both the number of proteins detected and quantification reproducibility when using SWATH-MS parameters modified from the original description [6]. However, these studies also concluded that the factors that impact detection by SWATH-MS are heavily dependent on both the hardware and the sample analysed and therefore should be empirically determined for different conditions.

Design of experiments (DoE) uses statistical methodology for identifying significant factors and then optimising a response by fine tuning them [11]. It is especially useful because it allows the study of a large number of candidate parameters with a minimal number of experiments; different combinations of variables are evaluated simultaneously, taking into account possible interaction effects. DoE has been widely used in other areas (for a review, see Hecht et al. [12]). However, its application to SWATH-MS has been limited, and in the absence of good data on critical parameters there remains a considerable ‘art’ to SWATH-MS acquisition.

The aim of this study was to maximize the number of proteins and peptides quantified per SWATH analysis on human serum samples without negatively impacting quantification precision. In order to do so, we evaluated the influence of different parameters and their interdependence, such as initial sample input, length of the LC separation, and several MS acquisition settings. We used a DoE approach, which is fully adaptable to other samples and instruments and may help other researchers optimize their workflows in a time and cost-effective manner. Moreover, we performed validation checks, testing the optimized method on raw and depleted samples.

## Methods

### Sample preparation

Human serum samples were collected from a healthy donor under informed consent and ethics approval (Understanding Mechanisms of Immune Mediated Disease project, Newcastle University). Blood was collected into Vacuette Z Serum Sep Clot Activator tubes (Greiner Bio One, Kremsmünster, Austria), and left to clot for a minimum of 30 min before being centrifuged at 2000×*g* for 10 min at room temperature. Serum was aliquoted and then stored at − 80 °C until use. Total protein content of crude and depleted serum samples was determined using the BradfordUltra Assay (Expedeon, Swavesey, UK) with bovine serum albumin (BSA) as standard. SDS-PAGE analysis of samples before and after depletion was carried out using the NuPAGE system (ThermoFisher, Waltham, MA, USA) using Bis–Tris gels (4–12%) combined with MOPS buffer (50 mM MOPS, 50 mM Tris Base, 0.1% SDS, 1 mM EDTA, pH 7.7). The gels were stained overnight with Coomassie solution (0.1% Coomassie Brilliant Blue R-250, 50% methanol, 10% glacial acetic acid, 40% H_2_O).

The twelve most abundant proteins in serum were depleted using Pierce™ Top 12 Abundant Protein Depletion Spin Columns (ThermoFisher, Waltham, MA, USA) according to manufacturer’s instructions. Briefly, 400 µg or 600 µg of total serum proteins were loaded onto the columns and incubated for 1 h at room temperature. Unbound low abundance proteins were recovered by centrifugation at 1000×*g* for 2 min.

The raw and depleted serum samples were mixed with cold acetone at 1:6 v/v ratio and precipitated at − 20 °C overnight, then centrifuged at 15,000×*g* for 15 min. After air-drying, the protein pellets were redissolved in 20 µL of 6 M urea, 100 mM Tris, pH8, reduced with 10 mM DTT for 1 h and alkylated with 35 mM iodoacetamide at room temperature in darkness for 1 h. Samples were then diluted with 50 mM NH_4_HCO_3_ to a final concentration of 1 M urea, and proteins were digested with sequencing-grade modified trypsin (Promega, Madison, WI, USA) at an enzyme–protein ratio of 1:100 (w/w) at 37 °C overnight. Digestion was terminated by adding 5% TFA, and then the peptides were desalted with homemade C18 cartridges (Supelco, Bellefonte, PA, USA), dried using a speed vacuum concentrator and diluted in 15 µL of mass spectrometry injection buffer (3% acetonitrile, 0.1% TFA). The same sample was used for all DoE experiments while five different ones were used for the method validation study.

### LC–SWATH MS setup

All the LC/SWATH-MS runs in this work were performed on a Dionex Ultimate 3000 RSLC nano-HPLC system connected to a TripleTOF 6600 mass spectrometer (AB SCIEX, Concord, Ontario) with a nano-electrospray ionization source. Each proteomic sample (∼ 1 μg) was loaded onto a PepMap 100 column (300 μm × 5 mm, 5 μm, 100 Å, ThermoFisher) using an isocratic flow of 97% buffer A (0.05% FA) and 3% buffer B (80% acetonitrile, 0.5% FA). Then, peptides were separated on a nanoLC column (75 μm × 23 cm) packed in-house with 3 μm resin (Dr. Maisch GmbH) at a flow rate of 400 nL/min.

Two different length elution gradients were used for SWATH-MS, a short one that went from 5 to 30% buffer B in 30 min and a long one that went from 5 to 30% buffer B in 90 min. All SWATH MS scans were acquired in high resolution mode and m/z-dependent rolling collision energy was applied with 5 V energy spread. The main SWATH acquisition parameters were set at different values following the DoE models (Table 1).

**Table 1:** SWATH acquisition parameters studied during the screening and optimization DoE experiments with upper and lower ranges explored

Experiment	SWATH acquisition parameters	Role	Lower range	Upper range
Screening design	Protein to deplete (µg)	Continuous	400	600
	Gradient length (min)	Continuous	30	90
	MS/MS accumulation time (ms)	Continuous	50	250
	Number of SWATH windows	Continuous	20	83
	MS mass range (m/z)	Categorical	400–800	350–1200
	MS/MS mass range (m/z)	Categorical	400–1200	100–1800
	SWATH window width	Categorical	Fixed	Variable
Optimization design	MS/MS accumulation time (ms)	Continuous	20	60
	Number of SWATH windows	Continuous	60	100

See text for details

### SWATH MS data extraction

Peaks were extracted from the raw SWATH data using the OpenSwathWorkflow (OpenMS 2.1.0) [13] for all data analyses. First, SWATH-MS.wiff files were centroided and converted to mzML using the SCIEX MS Data Converter 1.3. OpenSwath parameters were: min_rsq: 0.90, min_coverage: 0.6, min_upper_edge_dist: 1, mz_extraction_window: 30 ppm, rt_extraction_window: 300, extra_rt_extraction_window: 100. Peptides were identified by comparison with a SWATH assay library containing a compendium of the human plasma proteome (ProteomeXchange PXD001064) [14]. We chose a well characterized public library to maximise the reproducibility of the DoE work. Our rationale was that a published SWATH library using retention time alignment based on endogenous plasma peptides as we describe it is preferable to using a sample-specific library that is built on the same data set that is being analysed. The latter may give slightly higher hit rates, but the aim of this work was to generate a robust workflow comparable across many labs. Generating a sample-specific library would limit the reproducibility in other laboratories. Although the public library is based on a 5600 machine, we note that the 5600 and 6600 data align well in benchmarking studies. The final library contains more than 1600 human plasma proteins. RT was aligned using 10 endogenous plasma peptides.

The analysis workflow followed the standard recommended computational workflow—OpenSWATH, PyProphet, TRIC, IPF and TAPIR—see the full walkthrough at http://openswath.org/en/latest/index.html. In particular, note that we used an FDR cut-off of 10% for quality before applying an FDR cut-off of 1% on the proteins. The max quality FDR is an extension of the M-score cut off, meaning a peak group of this score will be considered for alignment. The target FDR is then applied to all said candidates to provide an overall 1% FDR. In SWATH2Stats there is again an FDR filter of 1% applied, this corresponds to the q value resulting in the matrix with 1% FDR at transition level. Thus OpenSWATH results were statistically validated using pyprophet 0.24.1 [15] with settings -d_score.cutoff = "1" –ignore.invalid_score_columns, and aligned using TRIC (msproteomicstools 0.7.0) [16] with the following parameters: –method LocalMST –max_rt_diff 60 –target_fdr 0.01 –max_fdr_quality 0.1 –mst:useRTCorrection True –mst:Stdev_multiplier 3.0 –alignment_score 0.005 –realign_method lowess –matrix_output_method full –dscore_cutoff 1.0 –frac_selected 0 –disable_isotopic_grouping. With TRIC alignment enabled there were no missing values and the analysis was performed on a complete data matrix. We note that not using TRIC alignment would generate missing values making the downstream DoE work difficult without resorting to imputation methods. The R package SWATH2Stats [17] was used to filter, annotate and export the aligned SWATH data into a readable format and initial graphs created in R using ggplot2 [18].

### Design of experiments

An initial resolution IV, fractional factorial screening design was used to determine which of the seven parameters studied (protein amount to deplete, gradient length, MS m/z range, MS accumulation time, MS/MS m/z range, fixed/variable windows and number of SWATH windows) affected the number of proteins quantified. The factors and experimental ranges (minimum and maximum levels) were selected based on previous experience and a literature review [19] followed by a brainstorming and prioritization exercise with scientific and technical staff. The MS/MS accumulation time was set so the maximum cycle time was 3.4 s—see Table 1.

Following the initial screening design, two critical parameters were selected to complete optimization using a face-centered Central Composite Design (CCD) with uniform precision. This permitted maximization of both the number of proteins quantified and quantification reproducibility. The number of SWATH windows and MS/MS accumulation time were evaluated on three levels: the minimum and maximum values in Table 1 plus centre-points testing for linearity and reproducibility. The remaining six, non-critical parameters from the initial experiment were set at their best level as determined by the screening design.

JMP® Pro 14 from SAS Institute Inc. (Cary, NC, USA) was used to create the designs and to evaluate the results. Blocking was included to account for potential nuisance factors and minimize potential risks due to equipment or other technical difficulties during the conduct of the study. This relatively conservative design strategy, resulted in a complete DoE series of 16 MS runs conducted in two blocks of eight runs for the screening design, plus 28 MS runs for the CCD conducted in two blocks of 7 runs which was repeated.

All designs, experimental data and analysis code are available in the figshare public depository at https://figshare.com/s/f45d38c784cfdc4f725e permitting others to replicate the designs and statistical analysis workflows—see 10.25405/data.ncl.14711058.

## Results

### Screening design

**Fig. 1:**
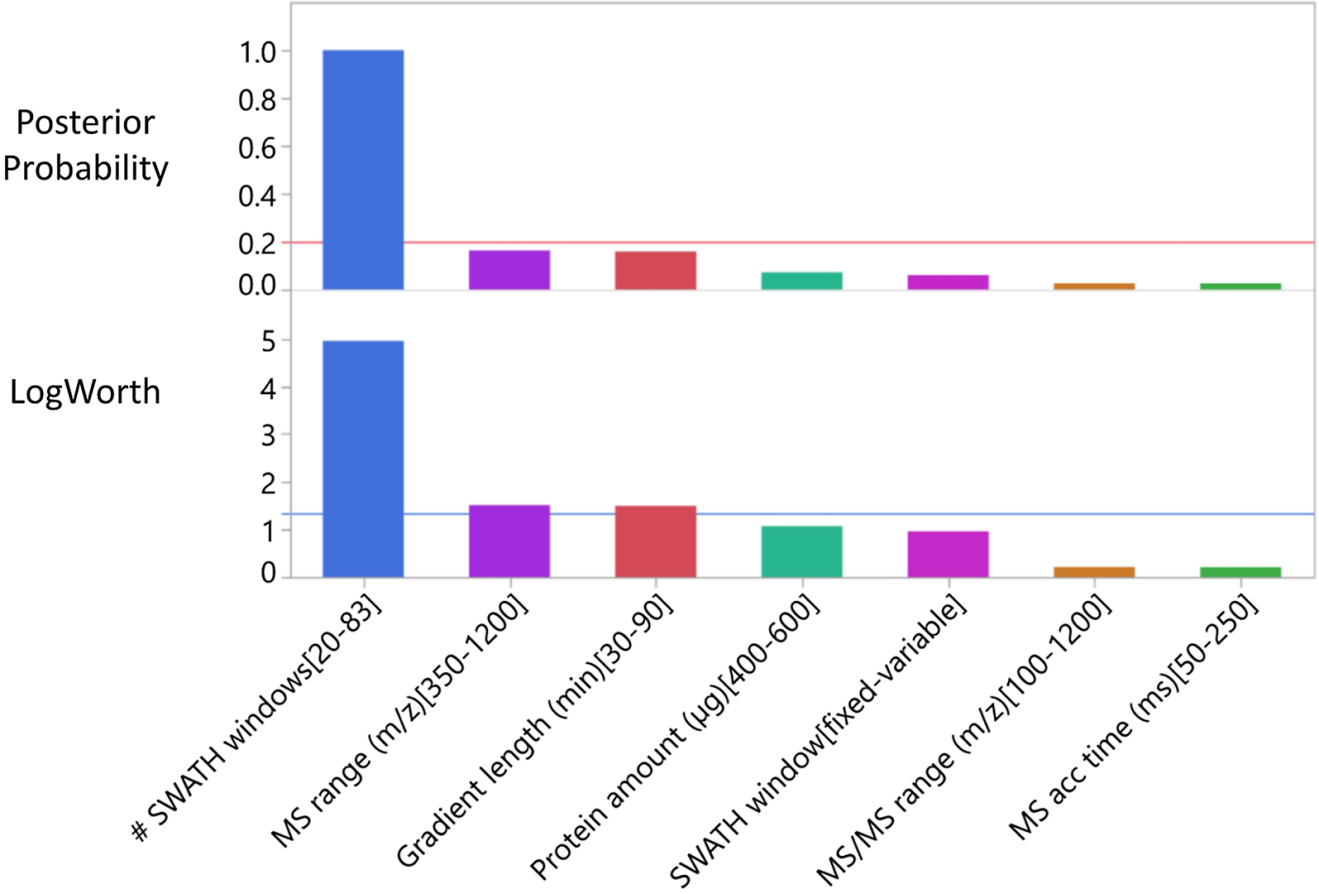
Bayes plot showing the posterior probabilities revised in light of the screening study data. The prior probabilities (p = 0.20) are indicated by the red horizontal line. Note the large increase in probability for the number of SWATH windows. The LogWorth value corresponding to a statistically significant effect at the p < 0.05 level of significance is indicated by the blue horizontal line. While the Number of SWATH windows, MS range and Gradient length are all statistically significant, the posterior probability increases only for the Number of SWATH windows. Increasing the number of precursor windows from 20 to 83 increases the number of proteins recovered. Note that within the experimental ranges used the method is relatively robust to variation in protein amount, choice of fixed or variable window, MS/MS range and MS accumulation times

The influence of the seven LC–MS acquisition parameters on the number of proteins quantified was studied simultaneously using the fractional screening design (Additional file 1: Table S1). Critical parameters were identified using Bayes’ Method assigning uniform prior probabilities of 0.20 to each candidate, then revising the priors in the light of the data to estimate the posterior probabilities (see Fig. 1).

The number of SWATH windows was the most critical parameter with a highly significant (p < 0.0001) impact on the number of proteins quantified. Using 20 precursor selection windows we extracted 207.6 (SD ± 13.80) proteins. Increasing the number of precursor windows to 83 increased the mean number of proteins extracted to 346.1 with reduced variability (SD ± 6.01).

While the number of proteins was robust to other parameters, a 30 min gradient length resulted in 13.9% more proteins than a 90 min one independently of the other factors studied. Accordingly, in subsequent experiments, the gradient length was set at 30 min, and the remaining non-significant parameters were set to the level that produced the best response (Table 2). The most critical parameter—number of SWATH windows—was advanced to the robust optimization stage of the study.

**Table 2:** Final optimized SWATH acquisition method

Factor	Level	Critical
Protein amount	400 µg	No
Gradient length	30 min	No
MS acc. time	50 ms	No
MS range	400–800 m/z	No
MS/MS range	400–1200 m/z	No
SWATH windows	Variable	No
Number of SWATH windows	100	Yes
MS/MS accumulation time	40 ms	Yes

The number of SWATH Windows and MS/MS accumulation time were chosen based on the CCD results. The 6 non-significant parameters were set to the level that produced the best response in the screening design

### CCD optimization

**Fig. 2:**
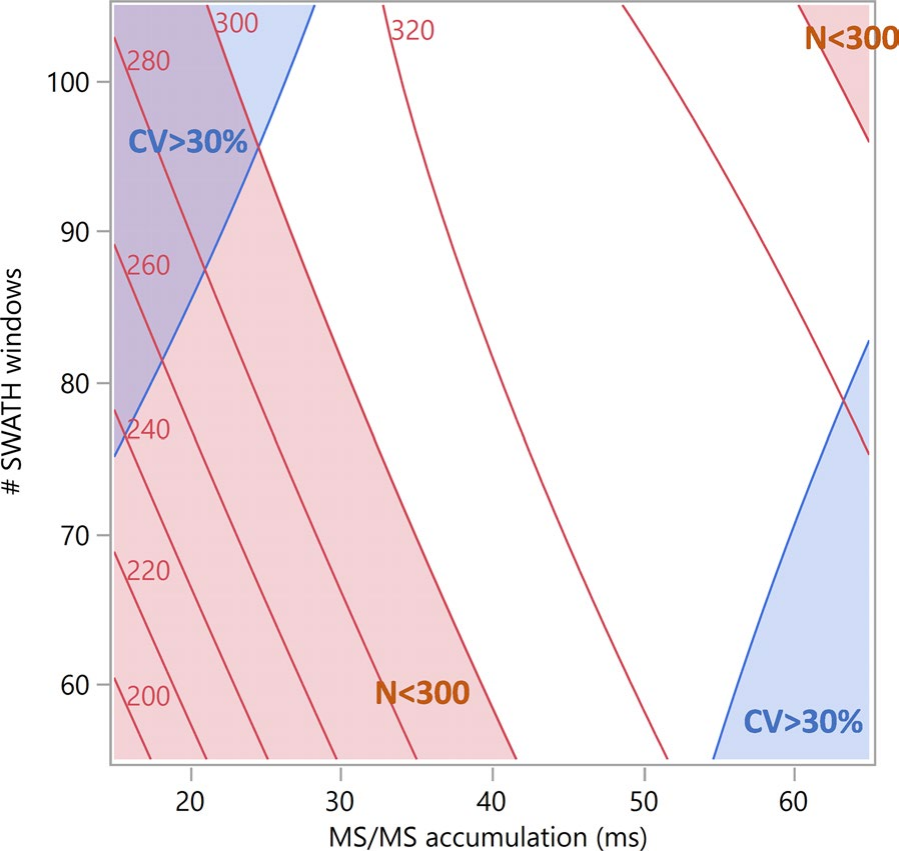
Contour plots showing the mean number of proteins (in red) as a function of the Number of SWATH Windows and the MS/MS accumulation time. The blue regions indicate areas of high variability (coefficient of variation > 30%). The pink regions indicate areas of low yield (less than 300 proteins recovered). The remaining space (in white) marks a safe operating region where we are likely to meet both constraints recovering higher numbers of proteins with reduced variability. The goal of robust optimization is to identify such regions

In the screening study, all experiments were conducted with a set MS/MS accumulation time to constrain the cycle time to less than the purported optimum of 3.4 s. However, as accumulation time and number of SWATH windows are thought to impact reproducibility, both parameters were tested simultaneously during the CCD optimization. The goal was to identify levels of each parameter giving the highest number of proteins and minimizing the coefficient of variance (CV) on peptide identifications (Additional file 1: Table S2). While the CV is relatively constant across this space, there is wide variation in the number of quantifiable proteins across the design space (see Fig. 2). The response surface model predicts well the number of proteins quantified (R^2^ = 0.95, p = 0.0035) with no significant lack-of-fit (p > 0.20). As expected the number of SWATH windows is important, but Accumulation Time too had a significant effect (*p* = 0.0014) on the number of proteins quantified, and there was a critical interaction between Accumulation Time and the number of SWATH windows (p = 0.0165).

**Fig. 3:**
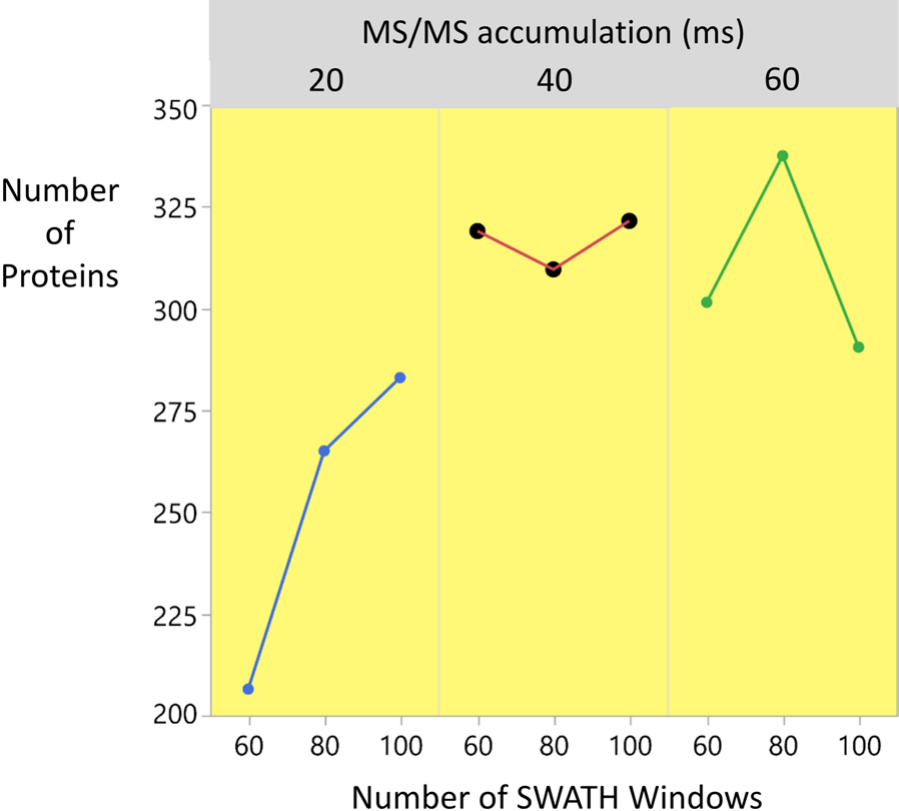
Mean number of proteins recovered as a function of MS/MS accumulation time and number of SWATH windows. More than 300 proteins were recovered at settings of both: 80 SWATH windows and an Accumulation time of 60 ms; 100 SWATH windows and an Accumulation time of 40 ms. While the number of proteins was maximized at 80 SWATH windows and an Accumulation time of 60 ms, the choice of 100 SWATH windows and 40 ms for the accumulation time was more robust—giving consistently over 300 proteins—with a cycle time of 4 s permitting satisfactory peak integration

Figure 3 shows the mean number of proteins obtained as a function of Accumulation time and the number of SWATH windows. While the number of proteins observed was maximized at an Accumulation time of 60 ms and 80 SWATH windows, these were not the settings progressed to the validation stage. Instead, an Accumulation time of 40 ms was robust to the choice of the number of SWATH windows and a choice of 100 SWATH windows was more robust to variation in Accumulation time—see Fig. 3. These values maintain a cycle time of 4 s, giving a mass spectrometry sampling frequency sufficient to obtain satisfactory peak integration: to adequately sample a Gaussian peak with a base line width of 8α, a minimum of eight points per peak should be acquired [20].

### Validation study

**Fig. 4:**
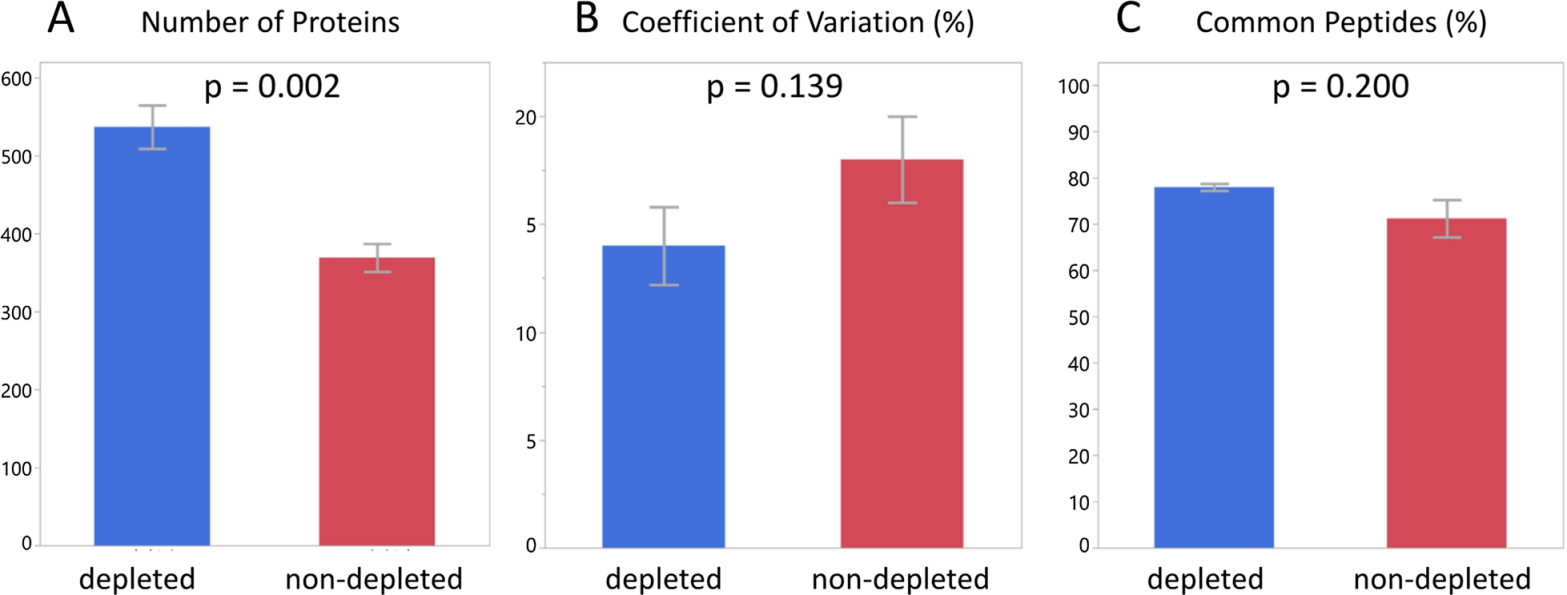
Means and standard errors for depleted (blue) and non-depleted (red) samples analysed using the optimized SWATH-MS method. Depletion has a significant effect on the number of proteins quantified (**A**) but not on the reproducibility between replicates as measured by the mean CV of peptide intensities (**B**) or the percentage of common peptides—those recovered in all replicates (**C**). See text for full explanation

We analysed 5 different serum samples in triplicate to verify our choice of robust settings (Table 2). In addition, we compared the results of raw samples and samples depleted of the most abundant proteins. Significantly more proteins were quantified in the depleted samples than in the non-depleted ones (see Fig. 4A) confirming previous observations for DDA experiments [21]. However, there was no significant difference in the reproducibility within replicates, as shown by the median CV of peptide intensities (Fig. 4B), or on the percentage of common peptides i.e. peptides detected in all 3 replicates (Fig. 4C).

## Discussion

Recent advances in MS technology permit large numbers of proteins to be quantified with good accuracy and reproducibility, making them an ideal tool for biomarker discovery [21–23]. One such technique is SWATH-MS which, theoretically, fragments all precursors present in a sample and acquires data in windows that cover, sequentially, the full range of m/z to be investigated. In consequence, the number of windows, the range they cover and the time allowed for fragment data accumulation, are some of the parameters that must be optimized to get the best results from a SWATH-MS experiment. In our study, we employed a two-step DoE approach to identify critical parameters and then build a model taking into account interactions and curvature to permit optimization in an economical number of MS experiments.

A full factorial (FF) design investigating every combination of the 7 initial factors minimum and maximum levels would provide the most information, however, it was rejected as it would require 128 MS runs. Without robotics this is logistically unfeasible. Instead a 16-run Resolution IV fractional factorial design was chosen instead. This design permits main effects and some 2-factor interaction effects to be estimated, even though they may be confounded with other 2-factor interactions, [11] while reducing the design to just 16 experiments by performing only a subset of the FF runs. We were able to create a model that successfully predicted the number of proteins that can be quantified with any combination of the parameters. Our results indicated that only the variation of the number of SWATH windows had a significant impact on this outcome—using more windows increased the number of quantified proteins. This is not surprising because increasing the number of windows while covering the same m/z range makes each window narrower, thus selecting fewer peptides for fragmentation and reducing ion interference, thereby facilitating peptide identification and increasing reproducibility. While the interaction effect was not statistically significant, the high number of windows may also be the reason why the best results were observed with a shorter LC gradient. Traditionally, longer gradients were necessary to separate peptides and increase their chances of fragmentation. However, a SWATH design with a high number of narrow windows is able to separately detect peptides with close elution times as shown in *E. coli* [24]. Similar results have been observed by other authors. Simbürger et al. [8] studied the effect of SWATH parameters on protein detection in lymphoma cell lines. They concluded that a high number of narrow windows increases both the number of quantified proteins and, to some extent, quantification reproducibility. A further increase of the window number yielded fewer proteins with only a slight improvement on reproducibility. This is due to the fact that, with constant cycle time, increasing the number of windows also decreases accumulation time which reduces sensitivity and the quality of the MS/MS spectra. Consequently, the optimum SWATH-MS method must find an equilibrium between number of windows and accumulation time. The next step of our DoE approach was to develop a model permitting identification of a robust operating region for the method.

Initial experimental data suggested that the relationship between windows and MS/MS accumulation time is not linear. Accordingly, we used a central composite design to model these non-linearities and capture their interaction while setting the rest of the parameters at those levels producing the best results in the screening design. Besides proteins quantified, three replicates were performed to calculate the impact on reproducibility. Optimization of number of SWATH windows jointly with accumulation time has already been shown to improve SWATH quantification performance on yeast samples [9]. When the number of windows is the same, a higher accumulation time improved quantification, as it increases sensitivity, but in our human serum samples this effect is limited. We found that a high accumulation time should be combined with a medium number of windows, or a medium accumulation time with both a low or high number of windows, for the parameters to be in equilibrium.

In this study, reproducibility measured as the CV of peptide intensities changed little across the parameter space. Previous studies have shown that CV was not impacted when different windows were used with the same accumulation time [24] or when different combinations of accumulation time and number of windows were examined, keeping a constant cycle time [8]. Furthermore, cycle time has no significant correlation with peptide CV as long as it allows for detection of 8 points per peak [9, 19]. For this reason, keeping a conservative (4 s) cycle time influenced our choice of optimum method as well as the number of proteins identified. In addition to optimizing our SWATH-MS results, we found that the designs allowed us to capture existing domain knowledge about SWATH-MS and adapt it to our samples and experimental set up. We found a design of experiments approach allowed us to map these complex multidimensional spaces in a highly efficient manner. As well as identifying and optimizing the two critical parameters, we obtained data demonstrating that the equipment is robust to variation in five other dimensions, improving the transferability and reproducibility of our research. The chosen method performed well in validation studies and gave considerable confidence in then analysing proteomics data for a large consortium study with multiple collaborators (manuscript in preparation).

## Conclusions

A structured, designed approach to SWATH-MS development permitted the identification of five parameters to which the method was relatively robust in addition to two process-critical parameters. Modelling the two critical parameters permitted identification of a robust operating window for the method maximizing both the number of proteins quantified and the reproducibility of the method. The resulting settings gave a high degree of confidence in the method prior to a large consortium study with multiple collaborators.

## Supplementary Information

**Additional file 1.** Supplementary statistical notes.

## Data Availability

All data generated or analysed during this study are included in this published article and its Additional files (see Additional file 1). Additional file 1: Tables S1 and S2 show the screening and CCD designs, runs, settings and proteins quantified. All designs, experimental data, and SAS JMP code are available in the figshare public depository at https://figshare.com/s/f45d38c784cfdc4f725e permitting others to replicate the designs and statistical analysis workflows—see 10.25405/data.ncl.14711058. The original mass spectrometry proteomics data are publicly available and have been deposited to the MassIVE Archive with the data set identifier MSV000085570. The authors thank Graham Smith, Nancy Woadden and Imogen Wilson for their assistance in data archiving. This work was supported by an MRC/Versus Arthritis funded stratified medicine award, Maximising Therapeutic Utility in Rheumatoid Arthritis (MATURA), Grant Number MR/K015346/1.

## Abbreviations

LC–MS: Liquid chromatography–mass spectrometry
DDA: Data-dependent acquisition
IDA: Information-dependent acquisition
MRM: Multiple reaction monitoring
DIA: Data-independent acquisition
SWATH: Sequential window acquisition of all theoretical spectra
DoE: Design of experiments
CCD: Central composite design
CV: Coefficient of variance
FF: Full factorial
QbD: Quality by design
