# Supplementary material for: Robust optimization of SWATH-MS workflow for human blood serum proteome analysis using a quality by design approach
Source: Clin Proteomics. 2021 Aug 12;18:20. doi: 10.1186/s12014-021-09323-z (PMC8359389; doi:10.1186/s12014-021-09323-z)
Supplement: Supplementary file 1 — Additional file 1. Supplementary statistical notes. [file 12014_2021_9323_MOESM1_ESM.docx]

# Additional file 1

| Pattern | Run | Block | Protein amount  (µg) | Gradient length  (min) | MS acc time  (ms) | # SWATH windows | MS range  (m/z) | MS/MS range (m/z) | SWATH window | Proteins  quantified |
| --- | --- | --- | --- | --- | --- | --- | --- | --- | --- | --- |
| +−++−+− | 1 | 1 | 600 | 30 | 250 | 83 | 400-800 | 400-1800 | fixed | 237 |
| ++−−++− | 2 | 1 | 600 | 90 | 50 | 20 | 350-1200 | 400-1800 | fixed | 96 |
| −++−−++ | 3 | 1 | 400 | 90 | 250 | 20 | 400-800 | 400-1800 | variable | 196 |
| −−−−−−− | 4 | 1 | 400 | 30 | 50 | 20 | 400-800 | 100-1200 | fixed | 145 |
| ++−+−−+ | 5 | 1 | 600 | 90 | 50 | 83 | 400-800 | 100-1200 | variable | 325 |
| +−+−+−+ | 6 | 1 | 600 | 30 | 250 | 20 | 350-1200 | 100-1200 | variable | 143 |
| −−−++++ | 7 | 1 | 400 | 30 | 50 | 83 | 350-1200 | 400-1800 | variable | 271 |
| −++++−− | 8 | 1 | 400 | 90 | 250 | 83 | 350-1200 | 100-1200 | fixed | 299 |
| −+−+−+− | 9 | 2 | 400 | 90 | 50 | 83 | 400-800 | 400-1800 | fixed | 371 |
| +++−−−− | 10 | 2 | 600 | 90 | 250 | 20 | 400-800 | 100-1200 | fixed | 180 |
| +++++++ | 11 | 2 | 600 | 90 | 250 | 83 | 350-1200 | 400-1800 | variable | 300 |
| −−++−−+ | 12 | 2 | 400 | 30 | 250 | 83 | 400-800 | 100-1200 | variable | 299 |
| +−−++−− | 13 | 2 | 600 | 30 | 50 | 83 | 350-1200 | 100-1200 | fixed | 237 |
| −+−−+−+ | 14 | 2 | 400 | 90 | 50 | 20 | 350-1200 | 100-1200 | variable | 166 |
| −−+−++− | 15 | 2 | 400 | 30 | 250 | 20 | 350-1200 | 400-1800 | fixed | 135 |
| +−−−−++ | 16 | 2 | 600 | 30 | 50 | 20 | 400-800 | 400-1800 | variable | 159 |

**Table S1**. Resolution IV Fractional Factorial Screening design conducted as two blocks of eight runs. Seven factors were analysed simultaneously as a series of runs at two levels to study their impact on the number of proteins quantified.

| Pattern | Run | Block | MS/MS accumulation  time (ms) | # SWATH windows | Proteins - mean quantified | Average peptide  % CV |
| --- | --- | --- | --- | --- | --- | --- |
| 00 | 1 | 1 | 40 | 80 | 304 | 50.28 |
| +− | 2 | 1 | 60 | 60 | 301.5 | 53.98 |
| −− | 3 | 1 | 20 | 60 | 206.5 | 25.70 |
| 00 | 4 | 1 | 40 | 80 | 312 | 18.89 |
| 00 | 5 | 1 | 40 | 80 | 313 | 22.07 |
| −+ | 6 | 1 | 20 | 100 | 283 | 32.19 |
| ++ | 7 | 1 | 60 | 100 | 290.5 | 43.07 |
| 0a | 8 | 2 | 40 | 60 | 319 | 20.63 |
| A0 | 9 | 2 | 60 | 80 | 337.5 | 8.21 |
| 00 | 10 | 2 | 40 | 80 | 318 | 17.25 |
| 0A | 11 | 2 | 40 | 100 | 321.5 | 28.21 |
| 00 | 12 | 2 | 40 | 80 | 314 | 36.86 |
| 00 | 13 | 2 | 40 | 80 | 296.5 | 36.40 |
| a0 | 14 | 2 | 20 | 80 | 265 | 49.23 |

**Table S2**. Face-Centred Central Composite Design blocked in two runs with three centre points in each block for optimization of SWATH-MS method. MS/MS accumulation time and number of SWATH windows were studied on three levels to find their optimum levels to increase number of proteins quantified and experiment reproducibility. Each run was performed in duplicate.

Additional Statistical Notes

1. Further technical details of the screening and response surface designs used in this study can be found in Montgomery DC. *Design and analysis of experiments*. John Wiley & Sons, 2017. Further details about QbD can be found in Schlindwein, WS & Gibson, M *Pharmaceutical Quality by Design: A Practical Approach*. John Wiley & Sons, 2018.
2. Note that the number of proteins was analyzed with, and without, the square root transformation for counts data. Results presented are based on the untransformed data.
3. The design settings and data are shown in **Tables S1** and **S2**.
4. In the screening design, estimates were screened using conventional t-tests of the effect estimates and standard errors, followed by Bayes plots with uniform prior probabilities – see Box GE, Meyer RD. An analysis for unreplicated fractional factorials. Technometrics. 1986 Feb 1;28(1):11-8. Both methods confirmed the critical importance of the number of SWATH Windows in determining the number of proteins quantified.
5. The response surface design: 1) confirmed the importance of the number of SWATH Windows; 2) identified significant curvature in MS/MS accumulation time with the number of proteins first increasing and then decreasing with increasing MS/MS accumulation time; and 3) flagged a significant interaction between the number of Swath Windows and MS/MS accumulation time.
6. All designs, experimental data and analysis code are available in the figshare public depository at <https://figshare.com/s/f45d38c784cfdc4f725e> permitting others to replicate the designs and statistical analysis workflows –<https://doi.org/10.25405/data.ncl.14711058> upon publication.
